# Waste Disposal Practices in Low-Income Settlements of South Africa

**DOI:** 10.3390/ijerph18158176

**Published:** 2021-08-02

**Authors:** Lorren Kirsty Haywood, Thandi Kapwata, Suzan Oelofse, Gregory Breetzke, Caradee Yael Wright

**Affiliations:** 1 Smart Places, Council of Scientific and Industrial Research (CSIR), Pretoria 0001, South Africa; soelofse@csir.co.za; 2 Environment and Health Research Unit, South African Medical Research Council, Johannesburg 2094, South Africa; thandi.kapwata@mrc.ac.za; 3 Department of Environmental Health, Faculty of Health Sciences, University of Johannesburg, P.O. Box 524, Auckland Park 2006, South Africa; 4 Unit for Environmental Science and Management, North West University, Pothchefstroom 2502, South Africa; 5 Department of Geography, Geoinformatics and Meteorology, University of Pretoria, Pretoria 0002, South Africa; greg.breetzke@up.ac.za (G.B.); caradee.wright@mrc.ac.za (C.Y.W.); 6 Environment and Health Research Unit, South African Medical Research Council, Pretoria 0001, South Africa

**Keywords:** environmental health, pollution, poverty, service delivery, waste management

## Abstract

Domestic solid waste is rapidly increasing due to accelerated population growth and urbanization. Improper waste disposal poses potential health risks and environmental concerns. Here, we investigated waste disposal practices in relation to household/dwelling characteristics in South African low-income communities. Data for 2014 to 2019 from a community-orientated primary care program were analyzed using logistic regression. Families who reported living in a shack were more likely to dump waste in the street. Households who reported using non-electric sources of fuel for heating/cooking, those who lacked proper sanitation, and those who did not have access to piped water inside the dwelling were more likely to dispose of waste by dumping it in the street/in the yard or burying it. Families living in low-income settlements are at risk of solid waste exposure and this situation is exacerbated by poor access to piped water, proper sanitation, and electricity.

## 1. Introduction

In South Africa, municipal solid waste is rapidly increasing due to accelerated population growth, rapid urbanization, and economic activities, while municipal service delivery is struggling to keep up [1,2,3,4,5]. While this waste stream commonly includes business waste, building and demolition waste, and garden refuse, it is the accumulation of domestic waste within settlements that has consequences on the health of people living predominantly in low-income communities [5,6].

It is the responsibility of government municipalities to implement effective waste management services including the removal, storage, transportation, and final disposal of waste [7,8]. The South Africa national standards for waste collection acknowledge different levels of service delivery, depending on the practicality and cost-efficiency in a given geographic area [9]. However, waste removal frequencies are prescribed to avoid health hazards. For example, containers must be removed within 24-h of being reported full, but at least once a week to avoid fly breeding under South African climate conditions [10]. In addition, the National Policy for the Provision of Basic Refuse Removal Services to indigent households typically found in low-income settlements [10] enforce municipalities to provide waste weekly collection services to households even if they cannot afford to pay for the service. Despite this, service delivery often does not respond to the needs of the community, especially in low-income areas [11,12]. Furthermore, Abel [13] reports that small housing units including blocks of flats in high density/low-income areas with high numbers of occupants (often exceeding the maximum planned occupancy numbers) do not have space to store waste, which results in waste being dumped outside to keep the inside living areas clean. In the absence of a designated refuse area, the waste is dumped anywhere [13]. Moreover, land near un-serviced areas in South Africa is impacted due to extensive littering and illegal dumping in streets, public spaces, and vacant land [2,8,14,15]. This is in part attributed to the geographical challenges of the servicing of low-income communities where road access can be limited, there is high settlement density with poor spatial planning and layout of settlements, and where illegal land tenure complicates or prohibits the delivery of waste collection services [8]. The disparity in service delivery between different areas is another contributing factor. Some households choose not to leave their municipal-provided bin out for collection due to the risk of bin theft [11]. Instead, they resort to illegal dumping despite having access to regular collection services by the municipality [11].

Uncollected and/or illegally dumped soiled nappies, food waste, sand, gravel, paper, plastic packaging, metal, and glass contribute to several environmental impacts [1,16,17]. Drains clogged with waste cause flooding during rainy seasons. High temperatures and humid conditions increase the leachate generation potential of dumped organic waste, which directly affects ecosystems by penetrating the soil and contaminating groundwater [18,19]. Uncollected and/or illegally dumped waste leads to human health impacts. The stagnation of water in waste items attracts mosquitoes and other insects which breed and spread vector-borne diseases [1]. Food waste attracts flies, insects, rodents, and other vermin which act as vectors that spread infectious diseases [1].

In some instances, domestic waste is burned, which creates smoke that contains carbon monoxide, particulate matter, and nitrogen oxides, all of which are hazardous to human health [1]. UN-Habitat health data show that acute respiratory infections and diarrhea are significantly higher in children living in households without regular waste removal services and where dumping or burning solid waste in the yard occurs, compared to households that receive (and use) a regular waste collection service [18]. Solid waste is generated faster than any other environmental pollutants [20,21] and burning of it produces greenhouse gases that contribute to global warming [22].

In South Africa, low-income communities often comprise settlements characterized by a combination of formal (e.g., house, flat) and informal (e.g., shack) dwellings, as well as have socio-economic challenges such as poverty, tenure insecurity, and overcrowding [23,24,25]. Low-income communities’ existence is partly attributed to the economic and political environment of the country, accessibility to land, lack of finance, and cost of building material [26]. Together with poor urban planning and poor governance, these factors result in the geographical expansion of these communities [24,27].

Most low-income communities consist of different dwelling structures [28]. Formal houses are made of bricks while informal dwellings include shacks or traditional huts (i.e., dwellings not in compliance with current planning and building regulations). Some of the most common features of these communities include overcrowding, small sizes of houses, poor building standards, lack of basic urban design amenities, and inadequate supply of municipal services including the supply of water and electricity, sewage collection/sanitation, and waste removal [29]. Areas such as those with these characteristics where communities live are characterized by UN-Habitat as “slums” [30].

Household and dwelling characteristics are underlying factors that affect human health and well-being [25,31,32,33]. The relationship between household characteristics (e.g., the main source of fuel used for cooking, the main source of water), dwelling characteristics (e.g., type of roof, floor type, presence of ceiling, windows, etc.) and waste management has been poorly investigated in the low-income community context. A 2011 study considered the strain posed by the lack of municipal services on public health in dense, low-cost housing communities in Cape Town [32]. Of the four communities living in low-cost housing who were surveyed, 68% said that they did not have waste bins inside their dwellings and 22% of the households disposed of their solid waste on the street. All the respondents in the survey complained of pests including rats, cockroaches, fleas, and flies carrying potential health risks within their immediate home environment [32].

Given the potential relations between household and dwelling characteristics and waste management, as well as the possible environmental health risks of uncollected waste on individuals living in low-income communities, this study investigated waste disposal practices, e.g., formal waste collection, illegal dumping (e.g., in street, open land, etc.), burying or burning of waste, in relation to household and dwelling characteristics among individuals living in low-income communities in South Africa. Gaining an understanding of these relationships is needed to motivate local municipalities to address waste management service delivery to all settlement areas and for all households, regardless of dwelling type, within their areas of jurisdiction. Such waste disposal practice information will also help inform the development of awareness campaigns for waste disposal best practices that protect public health.

## 2. Materials and Methods

### 2.1. Data Sources and Study Area

**Figure 1:**
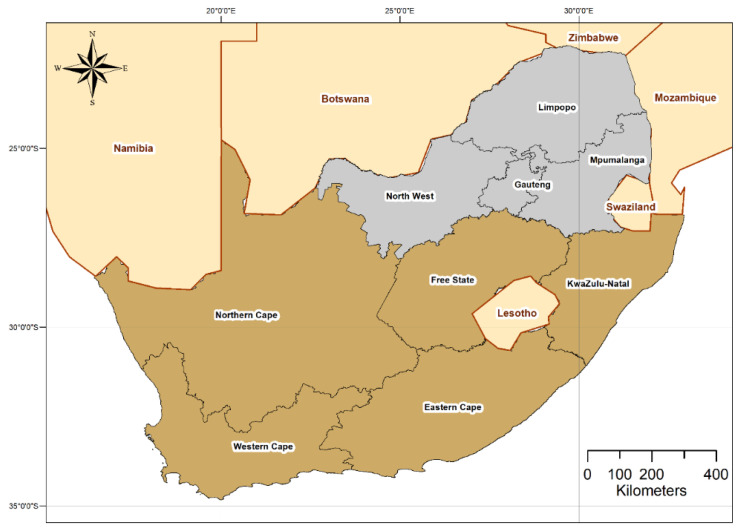
Map of South Africa indicating the provinces and major cities. The provinces for which data were applied in the analyses in this study are shaded in grey.

This was a secondary analysis of data collected by AitaHealth. AitaHealth is a mobile community healthcare management application available at https://mezzanineware.com/digital-productivity-technology/healthcare-technology-solutions/mobile-medical-assessment-app/ (accessed on 29 June 2021) and developed by the University of Pretoria’s Department of Family Medicine and Mezzanineware. The study areas where data were collected were areas with high numbers of people living in poverty, and not necessarily representative of the whole population in the provinces where community-oriented primary care (COPC) is implemented. For the purposes of these analyses, data between 2014 and 2019 were drawn from all sites implementing COPC in four South African provinces (mainly in Gauteng, but also in Limpopo, Mpumalanga, and the northwest, bordering on Gauteng, hence neighboring communities were included but to a lesser extent) (Figure 1).

Research ethics clearance for the study was granted by the University of Pretoria (102/2011) and permissions to use the dataset were in place.

### 2.2. Statistical Analysis

Logistic regression was used to assess relationships between dwelling characteristics, socio-demographic factors, and environmental health variables (i.e., fuel source, water supply, sanitation) and five waste disposal methods. The latter were: (i) burying or (ii) burning refuse in the yard; (iii) dumping refuse in the yard or (iv) in the street; (v) refuse being collected by a local authority once a week. The outcome was defined as binary with a negative response for each of the first four methods of disposal being coded as 0 and a positive response being coded as a 1 because improper waste disposal methods are environmental health risk factors. For the last method of disposal, a positive response was coded as 0 because it is assumed that regular waste collection is protective of human health whereas a negative response was coded as 1. All analyses were done using Stata Statistical Software version 15 [34] and *p* < 0.05 was considered as statistically significant.

## 3. Results

### 3.1. Sample Descriptive Results

Data for 89,411 households were analyzed. The majority were in Gauteng province (*n* = 80,846) with fewer in the northwest (*n* = 29), Mpumalanga (*n* = 23), and Limpopo (*n* = 11). The characteristics of these households and dwellings are reported in Table 1. A total of 54% of the sample lived in houses and flats considered “formal housing” while one-third of housing structures were considered informal dwellings referred to as shacks/huts (34%). Shared living quarters were difficult to decipher as formal versus informal. Informal dwellings were typically built from materials such as plastic, corrugated iron, wood, and cardboard.

From participants’ responses, about 30% of all the housing structures were not connected to a sewerage system. About half of the respondents stated that there was one or two people living in the dwelling. Unemployment was greater than 90%. Many households used non-electric sources of fuel (63%) with paraffin (31%) and wood (13%) being the most common main domestic fuel source for cooking/heating.

### 3.2. Patterns of Waste Disposal

Although the majority of households reported that their waste was collected once a week by the local authority, some households reported burning waste (5%), burying waste (3%), or dumping waste (3%) in the yard while 10% of households reported dumping waste in the street (Table 2).

### 3.3. Dwelling Characteristics and Waste Disposal

Statistically significant associations were found between households living in shacks/huts (OR = 4.9, 95% CI: 4.0–6.0, *p* < 0.001) or flats (OR = 4.7, 95% CI: 3.2–6.8, *p* < 0.001) and them reporting that they dump waste in the street (Table 3).

Households living in shacks, huts, and flats, compared to other housing types, were less likely to burn refuse in the yard and these associations were statistically significant (see Table 3).

### 3.4. Dwelling Characteristics and Waste Disposal

Households that said they used paraffin as their main fuel for cooking and/or heating (OR = 1.7, 95% CI: 1.5–2.1, *p* < 0.001) and made use of pit toilets (OR = 11.1, 95% CI: 9.24–13.36, *p* < 0.001) were reportedly most likely to dispose of refuse by dumping it in the street.

The reported use of wood as a domestic fuel for cooking and/or heating (OR = 6.5, 95% CI: 5.18–8.15, *p* < 0.001) and pit toilets (OR = 1.8, 95% CI: 11.19–17.17, *p* < 0.001) were statistically significant predictors of burning refuse in the yard. These were also statistically significant risk factors for both burying and dumping refuse in the yard (see Table 3).

Households who reported that their water sources were not within the dwelling structure (i.e., they were piped water from taps situated outside the yard, including standpipes, tanks, and streams) also stated that they tend to burn waste in the yard and/or communal waste burning areas.

Respondents reporting a lack of weekly refuse collection by local authorities also stated that their water sources were not within the yard (i.e., piped water outside the yard (OR = 1.3, 95% CI: 1.1–1.4, *p* < 0.001) and that they had to source water from tanks (OR = 1.4, 95% CI: 1.1–1.7, *p* = 0.001).

## 4. Discussion

**Figure 2:**
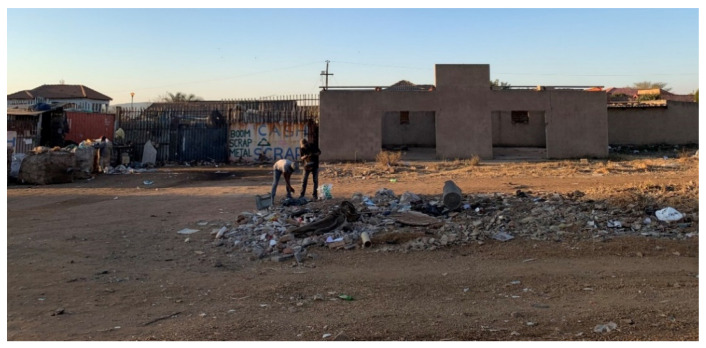
Waste dumped on the side of the street in an informal settlement.

Among a large sample of households living in low-income communities, solid waste was reported by many respondents as being collected on a weekly basis by the municipality, and yet up to 10% of households said they disposed of waste in other ways, such as dumping in the street (Figure 2)/yard, and/or burning or burying waste in the yard. Several reasons for these anomalies may exist despite a formal waste management system existing. For example, other studies suggest that formal waste collection can be erratic in low-income communities and despite weekly collection being mandatory by the government, it does not always occur weekly [4,35]. Reasons for this include a lack of resources or capacity within municipalities and challenges with suppliers [36].

**Figure 3:**
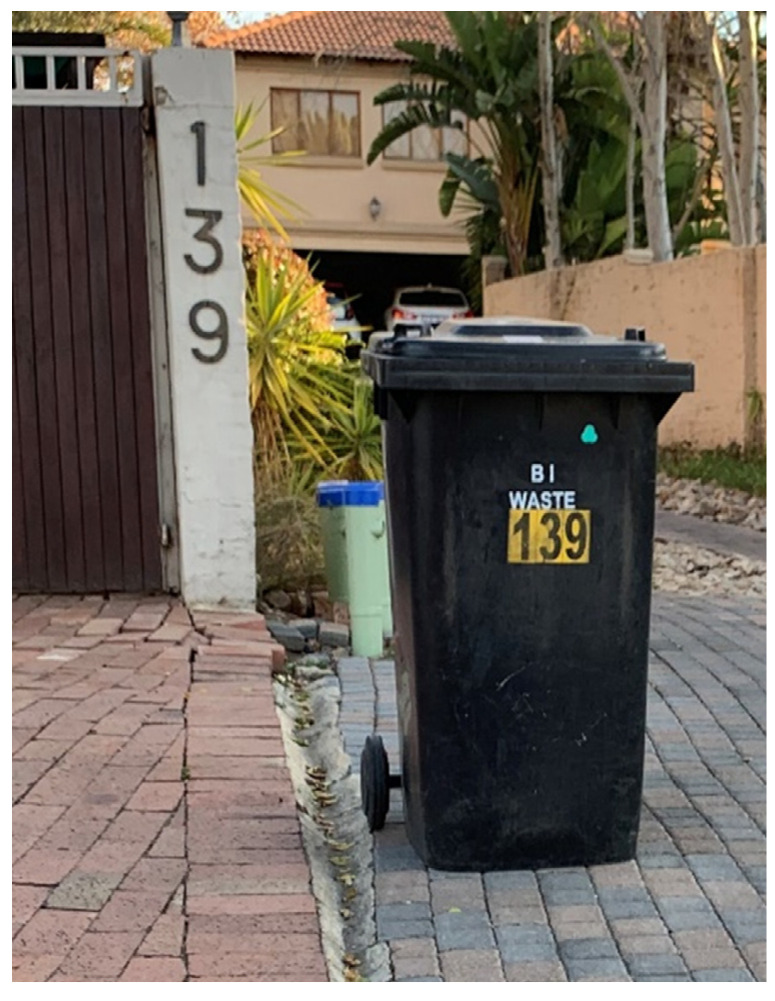
Illustration of a municipal-issued wheelie bin to a formal dwelling.

Also, the municipality typically issues a “wheelie bin” (Figure 3), or two plastic bin liners (black bags) per week, to households linked to a municipal account number and tenants as well as backyard dwellers (i.e., second house built on same property) are therefore excluded [11]. The bins are prone to theft, especially if the nearby community receives bin liners only or no service. Moreover, bins are not typically issued to informal dwellings (i.e., shacks) in low-income settlements. In addition, bins are issued based on the legal number of occupants per building, whereas overcrowding is high in low-income areas [11].

Formal housing in low-income areas in Johannesburg often includes informal shacks as backyard dwellings. Therefore, waste bins supplied by the municipality in low-income areas are generally insufficient to handle the amount of waste generated, thus leading to the need for alternate methods of waste disposal besides reliance on formal waste collection. In some low-income communities, the household waste removal service comprises communal skips that are placed in easily accessible locations for trucks to collect the skips and remove the waste in areas. Households are expected to take their waste to the communal collection point which may be far away, thereby possibly limiting the use of the communal skip.

According to the 2018 General Household Survey [37] 3.9% (*n* = 190,476) households in Gauteng make use of their refuse dump (possibly on-site/in the yard), while 2% (*n* = 97,680) of households dump or leave waste “anywhere”. Considering that low-income households generate between 17.2 and 22.6 kg of household waste per week [38], approximately 5763 tons of waste per week is not collected leaving 288,156 Gauteng households potentially at risk of exposure to domestic solid waste because of improper waste management practices. Media reports dating back to 2011 refer to giant rats roaming the streets of Alexandra, a low-income area in Johannesburg. The rats feed on discarded food waste and place people, especially babies and the elderly, at risk of contracting rat-bite fever, other diseases, and even death [39,40,41].

Households living in informal dwelling types in low-income communities have been found to dispose of waste in inappropriate ways that would leave waste exposed and a potential public health risk [42]. In our study, results showed a significantly significant relationship between the informal dwelling types, typically that of shacks/huts, and the dumping of waste in the street. This is of particular concern as children play in the streets and face the risk of exposure to, for example, feces via hand-to-mouth contact [43]. The burning of waste within the yard was reportedly less common, possibly due to a lack of yard space or because a communal site existed where community members burnt waste. The burning of waste, whether on a landsite, in a yard, or in a communal area is a form of air pollution that adversely affects human health and well-being [44].

Waste disposal along with the main household fuel source and type of sanitation available to the household provides an indication of the level of municipal services in the area. In general, results were indicative of communities with low socio-economic status, reflected by the high unemployment rates, and inequality. Therefore, a lack of adequate waste collection services and limited space to store and manage waste on-site was a likely explanation for waste being dumped in the street by shack and flat dwellers. Furthermore, waste dumped in the streets is meant to be cleaned up by the municipality based on their constitutional mandate of city cleansing [45]. Dumping locations where waste accumulates and open spaces that are not well maintained provoke people to dump waste, however, city officials have also identified that regular clearing of illegal dumping seems to attract more dumping in the same location [11].

Inherent inequality among households living in different dwelling types in low-income settlements, i.e., formal versus informal, affects access to basic services such as water, sanitation, electricity, and waste removal. This inequality is visible in waste management service delivery due to the high visibility of waste when it is not properly collected and/or managed. This is a significant issue for municipalities as the financial costs associated with addressing the environmental and health impacts of littering and illegal dumping are higher than the cost of developing and operating cost-effective and adequate waste management systems [19]. Littering is a major environmental problem and previous research confirms that households perceive littering and dumping of waste as an environmental problem that requires measures for control or eradication [46]. Our research emphasizes that socio-economic factors affect how households address their waste practices. There is a general understanding that waste is detrimental, but without the means to reduce and dispose of it, it will continue to impose financial, social, health, and environmental impacts.

The UN Habitat typically defines slum areas of low-income communities as those with a lack of adequate sanitation, potable water and electricity, lack of housing durability, and lack of security of tenure [30]. Less attention is given to issues of uncontrolled waste accumulation which we have shown occurs in low-income areas with formal and informal dwellings. Future research should consider solid waste removal in domestic settings with formal and informal dwellings and the role it may play in creating slum-like conditions in low-income settlements.

Our research has shown that a lack of waste collection by authorities leads to improper waste disposal that has the potential to pollute the environment and exposes communities to significant health hazards. Our findings provide evidence to policymakers that more needs to be done to achieve SDG-11 (Sustainable Development Goal 11) which states that waste collection and management are essential public services for every community and are necessary for the protection of public health and the environment. Furthermore, this study highlights that socio-economically disadvantaged communities such as dwellers of informal settlements are not only subjected to poor health outcomes due to inadequate housing but also due to the unhealthy environmental conditions that they are exposed to because of the lack of municipal waste services.

This study has several strengths and limitations. The sample size was sufficiently large to test for statistically significant relations. While the sample is large, the data were mostly representative of low-income communities in one province and the findings may not be directly transferrable to settlements in other provinces. The data were self-reported and therefore may be prone to recall bias, however, since waste collection is a weekly occurrence, it is less likely to be poorly reported.

## 5. Conclusions

Households living in low-income settlements in South Africa are at risk as a result of poor waste collection service delivery. This situation is further exacerbated by the lack of other appropriate municipal services including access to water, proper sanitation, and electricity. Ineffective and irregular waste collection services result in environmental pollution associated with waste practices such as burying, burning, or dumping of waste in the streets and in open spaces in close proximity to dwellings. The environmental impacts relate to soil, water, and air pollution, while the health impacts are associated with exposure to vector-borne diseases and air pollution. Unraveling the complexities of household waste disposal practices among a mix of formal and informal dwellings in low-income settlements, together with municipal waste collection and disposal will help inform municipal decision-making and public health campaigns to reduce exposure to uncollected waste.

## Figures and Tables

**Table 1:** Summary characteristics of households and dwellings for all households in the dataset. Percentage frequencies do not sum to 100% due to missing data.

Variables	Frequency (*n*)	Frequency (%)
Gender of head of household		
Female	51,241	57
Male	38,163	42
Type of home		
Collective living quarters	7967	10
Flat	2091	2
House	38,273	52
Hut/shack	25,028	34
Number of people living in dwelling		
1–2	8949	55
3–5	5708	35
6–10	1446	8
>10	86	0.5
Number of people employed in dwelling		
0	84,588	94
1–2	4306	4
≥3	517	0.5
Fuel used for heating/cooking		
Electricity	27,224	37
Candles	8628	11
Coal	279	0.3
Gas	3167	4
Paraffin	22,185	30
Solar	1695	2
Wood	9541	13
Toilet type		
Flush toilet connected to a sewage system	50,769	69
Flush toilet with septic tank	2350	3
Chemical toilet	1973	2
Bucket toilet system	1694	2
Pit toilet	16,161	22
Source of water in the home		
Piped water in house/yard	53,882	76
Piped water outside yard	11,035	15
Tank	4903	6
Stream	588	0.8

**Table 2:** Self-reported waste disposal characteristics for all households in the dataset. Percentage frequencies do not sum to 100% due to missing data.

Self-Reported Waste Disposal Characteristics for All Households in the Dataset	Frequency (*n*)	Frequency (%)
Waste disposal—collected by local authority once a week		
Yes	61,948	69
No	11,349	12
Do not know	110	0.1
Refuse to answer	19	0
Waste disposal—burn in the yard		
Yes	5161	5
No	67,553	75
Do not know	672	0.7
Refuse to answer	40	0
Waste disposal—bury in the yard		
Yes	3337	3
No	69,397	77
Do not know	668	0.7
Refuse to answer	24	0
Waste disposal—dumped in the yard		
Yes	3443	3
No	69,695	77
Do not know	258	0.2
Refuse to answer	29	0
Waste disposal—dumped in the street		
Yes	9472	10
No	63,332	70
Do not know	579	0.6
Refuse to answer	40	0

**Table 3:** Results of multivariate regression of housing characteristics, socio-demographic factors, and environmental health risk factors in relation to waste disposal methods.

Variable	Dumped in the Street			Burn in the Yard			Bury in the Yard			Dumped in the Yard			Waste Collected		
	*OR*	*95%CI*	*P-Value*	*OR*	*95%CI*	*p-Value*	*OR*	*95% CI*	*p-Value*	*OR*	*95% CI*	*p-Value*	*OR*	*95% CI*	*p-Value*
**Fuel used for heating/cooking**															
Electricity *															
Candles	0.6	0.4–0.8	0.004	3.0	2.3–3.9	<0.001	1.4	1.0–2.0	0.051	2.0	1.4–2.9	<0.001	1.0	0.8–1.2	0.521
Coal	1.4	0.5–3.9	0.520	2.4	0.7–7.8	0.117	4.7	1.5–14.0	0.006	3.1	0.8–10.9	0.076	1.4	0.6–3.1	0.359
Gas	0.9	0.6–1.4	0.773	1.1	0.6–1.9	0.629	1.5	0.8–2.7	0.144	1.3	0.7–2.4	0.404	0.8	0.6–1.0	0.185
Paraffin	1.7	1.5–2.1	<0.001	1.1	0.9–1.4	0.271	1.2	0.9–1.6	0.12	2.2	1.7–3.0	<0.001	1.0	0.8–1.1	0.962
Solar	1.4	0.9–2.1	0.076	1.9	1.2–3.0	0.002	2.2	1.3–3.6	0.002	1.7	1.0–3.1	0.052	1.2	0.8–1.6	0.219
Wood	1.4	1.1–1.7	0.001	6.5	5.1–8.1	<0.001	5.0	3.8–6.5	<0.001	5.0	3.8–6.7	<0.001	0.8	0.6–0.9	0.021
**Type of home**															
House *															
Flat	4.7	3.2–6.8	<0.001	0.1	0.0–0.5	0.002	0.5	0.2–1.3	0.164	0.2	0.0–0.8	0.021	0.9	0.7–1.2	0.591
Collective living quarters	1.7	1.2–2.3	<0.001	1.0	0.8–1.4	0.523	1.7	1.2–2.3	0.001	1.3	0.9–1.8	0.088	1.0	0.8–1.2	0.631
Hut/shack	4.9	4.0–6.0	<0.001	0.4	0.3–0.5	<0.001	0.5	0.4–0.6	<0.001	0.6	0.4–0.7	<0.001	1.1	0.9–1.2	0.238
**Toilet type**															
Flush toilet connected to a sewage system *															
Flush toilet with septic tank	3.1	2.1–4.7	<0.001	3.2	2.1–5.0	<0.001	7.0	4.6–10.7	<0.001	3.6	2.2–5.8	<0.001	1.2	0.9–1.6	0.193
Chemical toilet	2.6	1.9–3.6	<0.001	1.2	0.7–2.0	0.488	3.1	1.8–5.5	<0.001	0.8	0.3–1.9	0.706	1.1	0.8–1.5	0.46
Bucket toilet system	1.3	0.9–1.9	0.056	0.5	0.3–1.0	0.051	0.9	0.4–1.9	0.917	0.5	0.2–1.2	0.166	1.0	0.7–1.5	0.7
Pit toilet	11.1	9.2–13.3	<0.001	13.8	11.1–17.1	<0.001	11.7	8.9–15.3	<0.001	8.3	6.4–10.7	<0.001	0.9	0.8–1.1	0.647
**Source of water in the home**															
Piped water in house/yard *															
Piped water outside yard	2.2	1.9–2.6	<0.001	2.2	1.8–2.6	<0.001	2.3	1.9–2.9	<0.001	2.9	2.4–3.5	<0.001	1.2	1.1–1.4	<0.001
Tank	0.8	0.6–1.0	0.064	4.6	3.7–5.7	<0.001	1.6	1.2–2.1	<0.001	0.9	0.7–1.3	0.919	1.4	1.1–1.7	0.001
Stream	2.9	1.7–5.1	<0.001	2.7	1.5–4.9	0.001	7.3	4.3–12.4	<0.001	3.1	1.7–5.8	<0.001	0.9	0.4–1.7	0.763
**Number of people living in the home**															
1–2 *															
3–5	1.0	0.9–1.2	0.220	1.0	0.9–1.2	0.416	1.2	0.9–1.4	0.063	0.9	0.8–1.1	0.751	1.0	0.9–1.1	0.502
6–10	0.7	0.6–1.0	0.088	0.9	0.6–1.1	0.434	1.2	0.8–1.6	0.231	1.2	0.9–1.6	0.205	1.0	0.8–1.2	0.786
>10	0.6	0.1–2.3	0.507	0.7	0.2–2.2	0.664	1.0	0.3–3.9	0.895	0.5	0.1–2.5	0.453	0.6	0.2–1.5	0.370
**Number of people employed in the home**															
0 *															
1–2	1.1	0.7–1.5	0.585	1.39	0.9–2.0	0.108	0.9	0.6–1.5	0.890	0.6	0.4–1.0	0.066	0.8	0.6–1.0	0.118
≥3	0.2	0.1–1.3	0.108	0.47	0.0–3.8	0.485	0.5	0.0–4.1	0.517	0.8	0.1–4.2	0.861	1.3	0.5–3.3	0.474

Note. * means reference category and refers to the reference category in each variable. OR refers to Odds Ratio and 95%CI refers to 95% Confidence Interval.

## Data Availability

Restrictions apply to the availability of these data. Data were obtained from the University of Pretoria Department of Family Medicine and are available from the authors with the permission of the University of Pretoria Department of Family Medicine.
